# A Method for Fabricating Cavity-SOI and Its Verification Using Resonant Pressure Sensors

**DOI:** 10.3390/mi16030297

**Published:** 2025-02-28

**Authors:** Han Xue, Xingyu Li, Yulan Lu, Bo Xie, Deyong Chen, Junbo Wang, Jian Chen

**Affiliations:** 1 State Key Laboratory of Transducer Technology, Aerospace Information Research Institute, Chinese Academy of Sciences, Beijing 100190, China; xuehan20@mails.ucas.ac.cn (H.X.); lixingyu21@mails.ucas.ac.cn (X.L.); luyl@aircas.ac.cn (Y.L.); xiaobo@aircas.ac.cn (B.X.); dychen@mail.ie.ac.cn (D.C.); chenjian@mail.ie.ac.cn (J.C.); 2 School of Electronic, Electrical and Communication Engineering, University of Chinese Academy of Sciences, Beijing 100049, China

**Keywords:** Au–Si eutectic bonding, cavity silicon on insulator, device fabrication, microelectromechanical system, resonant pressure sensor

## Abstract

Cavity silicon on insulator (Cavity-SOI) offers significant design flexibility for microelectromechanical systems (MEMS). Notably, the shape and depth of the cavity can be tailored to specific requirements, facilitating the realization of intricate multi-layer structural designs. The novelty of the proposed fabrication methodology is manifested in its employment of a micromachining process flow, which integrates dry etching, wafer level Au–Si eutectic bonding, and chemical mechanical polishing (CMP) to create Cavity-SOI. This innovative approach substantially mitigates the complexity of fabrication, and the implementation of wafer-level gold–silicon eutectic bonding and vacuum packaging can be achieved, representing a distinct advantage over conventional methods. To evaluate the technical viability, a MEMS resonant pressure sensor (RPS) was designed. Experimental findings demonstrate that during the formation of Cavity-SOI, dry etching can accurately fabricate cavities of predefined dimensions, wafer-level Au–Si eutectic bonding can achieve efficient sealing, and CMP can precisely regulate the depth of cavities, thus validating the feasibility of the Cavity-SOI formation process. Additionally, when implementing Cavity-SOI in the fabrication of MEMS RPS, it enables the spontaneous release of resonators, effectively circumventing the undercut and adhesion issues commonly encountered with hydrofluoric acid (HF) release. The sensors fabricated using Cavity-SOI exhibit a sensitivity of 100.695 Hz/kPa, a working temperature range spanning from −10–60 °C, a pressure range of 1–120 kPa, and a maximum error of less than 0.012% full scale (FS). The developed micromachining process for Cavity-SOI not only streamlines the fabrication process but also addresses several challenges inherent in traditional MEMS fabrication. The successful fabrication and performance validation of the MEMS RPS confirm the effectiveness and practicality of the proposed method. This breakthrough paves the way for the development of high-performance MEMS devices, opening up new possibilities for various applications in different industries.

## 1. Introduction

**Figure 1:**
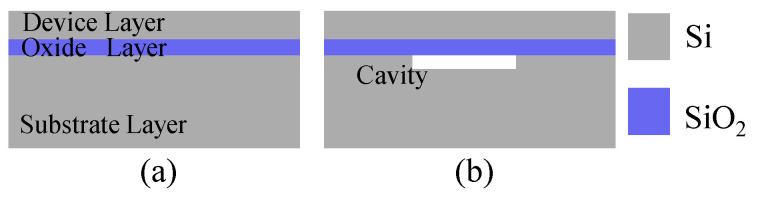
The schematic diagram of the structure. (**a**) Standard SOI, (**b**) Cavity-SOI.

Standard SOI is widely used in the fabrication of MEMS devices, such as pressure sensors [1,2], inertial sensors [3,4], microfluidic devices [5], and other MEMS devices [6]. It comprises a substrate layer as the base, a device layer commonly used for device fabrication, and a buried oxide layer in the middle, as shown in Figure 1a.

Cavity-SOI is a customized type of SOI. Similar to standard SOI, its structure also includes a substrate layer, a buried oxide layer, and a device layer, as depicted in Figure 1b. Specifically, there are cavities buried within the middle of the wafer [7]. Owing to the presence of these cavities on the substrate layer, Cavity-SOI offers several advantages in the manufacturing process of MEMS devices [8,9,10]. For instance, the number of fabrication steps can be reduced [11]. Simultaneously, it can avoid the undercut problem caused by hydrofluoric acid (HF) release when manufacturing movable components like pressure or inertial sensors [1,12,13].

Cavity-SOI is typically fabricated by bonding two silicon wafers. Prior to bonding, a silicon oxide layer is formed on the wafer surface via an oxidation process to serve as the SOI oxide layer. This layer acts as an insulator between the substrate layer and the device layer [14,15]. Cavity-SOI is a solution that meets the miniaturization and high performance requirements of semiconductor and MEMS technology. Lee et al. suggested that Cavity-SOI facilitates the packaging of pressure sensors [16]. Luoto et al. established design rules for Cavity-SOI manufacturing [17].

Currently, Cavity-SOI technology is utilized to fabricate a wide variety of high-performance MEMS devices. This technology not only allows for the production of devices with more intricate structures but also streamlines the manufacturing process. By fabricating the cavity in advance, it can reduce the number of etching steps and eliminate the release steps for movable parts [15,18,19].

However, the fabrication of Cavity-SOI using Si-Si direct bonding requires high temperature annealing at around 1000 °C to ensure the bonding quality [6,14,15]. This high temperature requirement may lead to compatibility issues with MEMS processes. Meanwhile, in the existing literature, the application of Cavity-SOI mainly achieves only mechanical connections, and vacuum packaging is rarely realized [17,18].

In this paper, we employed wafer level Au-Si eutectic bonding to fabricate Cavity-SOI. With this method, the bonding temperature can be reduced to 390 °C, and vacuum packaging can be achieved. Simultaneously, the CMP process can be utilized to fabricate device layers of any desired thickness, which offers greater flexibility in the fabrication process [12].

To verify the feasibility of the fabricated Cavity-SOI, we used it to fabricate MEMS RPSs. The experimental results indicate that the use of customized Cavity-SOI not only simplifies the fabrication process and resolves the undercut problem caused by HF release but also enables the RPSs to achieve comparable performance in terms of sensitivity and accuracy when compared with those fabricated using standard SOI [20,21,22,23].

## 2. Fabrication of the Cavity-SOI

**Figure 2:**
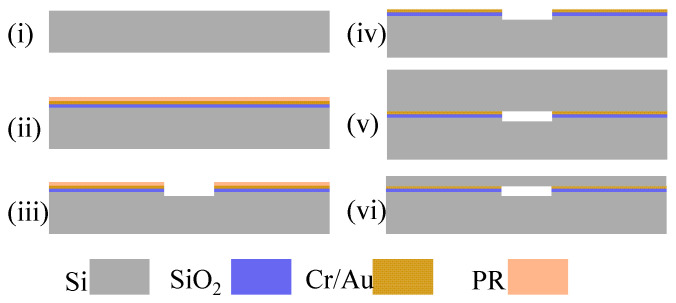
Fabrication steps of Cavity-SOI. (**i**) Clean the silicon wafer. (**ii**) Growth of an oxide layer as an insulating layer on the cleaned silicon wafer surface, followed by evaporation of a layer of Cr (50 nm) as an adhesive layer, a layer of Au (500 nm) as the bonding material, and spin coating of a layer of photoresist (PR) as a patterned mask. (**iii**) The cavities were etched on the surface of the silicon wafer with a depth of 10 μm. (**iv**) Clean the surface photoresist with acetone, alcohol, and deionized water. (**v**) Bond another silicon wafer at temperature of 390 °C and pressure of 2000 mbar. (**vi**) Thin the bonded silicon wafer to a predetermined thickness through CMP method.

The Cavity-SOI was fabricated by wafer-level Au–Si eutectic bonding of two silicon wafers and then thinning them to a specific thickness. Figure 2 shows the main fabricating steps, including cavity etching, eutectic bonding, and CMP.

First, a double-sided polished silicon wafer (4 inchs, 300 μm thickness, N-type, <100> crystal phase) was cleaned with concentrated sulfuric acid and deionized water as shown in Figure 2i. Then was deposited a layer of SiO_2_ by low pressure chemical vapor deposition on the surface of the wafer as an insulating layer. Then a 50 nm thick chromium layer and 500 nm thick Au layer were deposited by electron beam evaporation on the side of the wafer as an adhesion layer and bonding layer. Then a layer of photoresist (SPR220) was spin coated on the surface of the gold, as shown in Figure 2ii. And then photolithography and dry etching processes were used to fabricate the cavities on the surface of the silicon wafer, as shown in Figure 2iii. Then clean the wafer with acetone, anhydrous ethanol, and deionized water in sequence to remove the photoresist, as shown in Figure 2iv.

For the Au–Si eutectic bonding, the metallic layers needed for bonding were first deposited onto the silicon wafer with the etched cavity. The chromium–gold layer essential for eutectic bonding was initially created via an evaporation process. Then, it was bonded to a cleaned, double-sided polished silicon wafer (4 inchs, 300 μm thickness, N-type, <100> crystal phase) at 390 °C under a pressure of 2000 mbar [24], as shown in Figure 2v; the pressure and temperature were maintained for 30 min.

To achieve a good bonding effect, we need to carefully clean the two wafers to reduce surface particles and contamination. For the etched wafers, we first use acetone, absolute ethanol, and deionized water in sequence to clean and remove surface organic substances. Then they are cleaned in high temperature deionized water (95 °C, 10 min). For the un-etched wafers, concentrated sulfuric acid (90 °C, 15 min) was used to clean and remove surface particles and then cleaned by high temperature deionized water.

On the other hand, to reduce the impact of water vapor and other gases on bonding, during the bonding process, in the vacuum environment of the bonder, before applying pressure, the temperature needs to be first raised to 200 °C and held for 60 min to thoroughly dry the wafers. Then, the temperature is further increased to 390 °C for bonding.

After bonding, it was necessary to thin the bonded silicon wafer to obtain a device layer with a thickness of 40 μm for resonator fabrication, as shown in Figure 2vi. The CMP process consisted of three stages: first, initial thinning with a coarse abrasive; second, subsequent thinning with a fine abrasive to reach the desired thickness; and, finally, polishing with a polishing liquid to meet the bonding requirements. Through the above-mentioned processing steps, the Cavity-SOI was fabricated and can be used to process RPS.

The thickness of the Cavity-SOI was required to meet the specified value, with a total thickness variation not exceeding 10 μm. When we measured the Cavity-SOI thickness using a thickness gauge, the result showed a range of 338–343 μm.

**Figure 3:**
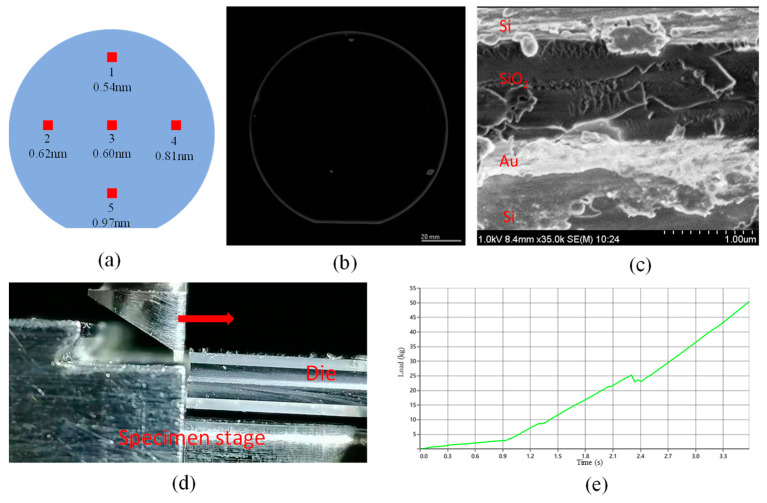
Characterization of CMP and bonding quality. (**a**) Measurement results of surface roughness, ranging from 0.54 to 0.97 nm. (**b**) Test results of the bonding using an ultrasonic scanning microscope. The light-colored areas represent unbonded regions. (**c**) Scanning electron microscope image of the bonding interface. (**d**) Testing of the bonding shear force using a push–pull tester. (**e**) Shear force test results. The bonding can withstand a shear force of over 50 kgf.

We used a step profiler to measure the surface of the sample after the CMP process. The measurement results are shown in Figure 3a. We evenly selected five points on the wafer surface for measurement. The surface roughness values obtained from these measurements were all less than 1 nm, indicating an extremely smooth surface finish.

To characterize the bonded silicon wafers, we used an ultrasonic scanning microscope, as shown in Figure 3b. The characterization results clearly showed that the bonding quality was satisfactory. Although there were only very small unbonded areas in a few specific regions, these areas could be attributed to the presence of dust particles and contaminants on the bonding surfaces before bonding. Proper cleaning of the bonding surfaces before the bonding process can effectively reduce both the number and the area of such unbonded regions.

Moreover, we employed a scanning electron microscope to examine the bonding interface, as presented in Figure 3c. The microscopic images indicated that there were no observable holes at the bonding interface, which further verified the excellent bonding quality achieved in this study.

As depicted in Figure 3d, the bonded wafer was diced into 5 mm × 5 mm square pieces. Subsequently, a push–pull tester was employed to measure the shear force of the bonding interface. The obtained test results are illustrated in Figure 3e. The bonding surface demonstrated the capability to endure a shear force exceeding 50 kgf. Through calculations, it was determined that the bonding could withstand a shear stress of more than 1.96 × 10^7^ Pa. These findings provide evidence for the reliability of the bonding process.

## 3. Verification of the Cavity-SOI

### 3.1. Sensor Design

**Figure 4:**
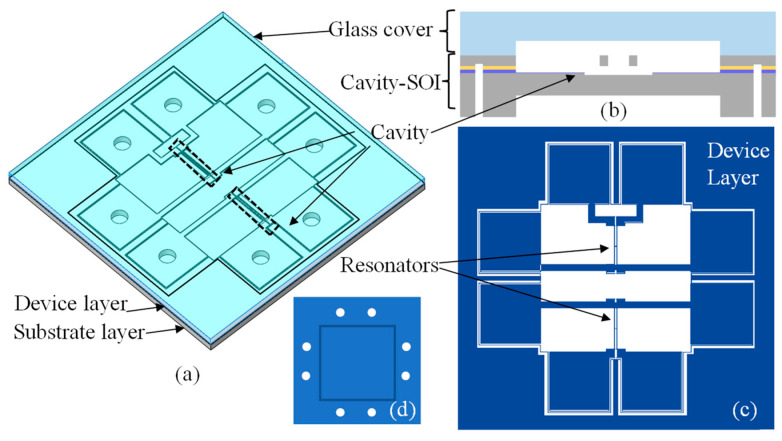
Design of the pressure sensor. (**a**) Schematic diagram of the overall structure of the sensor, which consists of a glass cover plate and a Cavity-SOI from top to bottom. (**b**) Schematic diagram of the sensor structure; the cavity is located directly below the resonator (**c**) Schematic diagram of the device layer, where resonators are located at different positions on the device layer and connected to the pads at the edges through silicon wires. (**d**) Schematic diagram of the substrate layer.

Since the Cavity-SOI was fabricated for use in the MEMS RPS, the design of the Cavity-SOI should be in accordance with the design of the sensors. The schematic diagrams of the pressure sensor are presented in Figure 4a,b. The sensor chip is composed of two main components: the Cavity-SOI fabricated previously and a cover made of BF33 glass (SCHOTT, Suzhou, China).

Two cavities were fabricated on the substrate layer of the Cavity-SOI. The resonators were fabricated on the device layer, as illustrated in Figure 4c. Meanwhile, a diaphragm (with a length of 5000 μm, a width of 5000 μm, and a thickness of 120 μm) and electrode lead holes (with a diameter of 300 μm) were fabricated on the back side of the substrate layer, as shown in Figure 4d. The thickness of the device layer of the Cavity-SOI was designed to be 40 μm, and the thickness of the substrate layer is 300 μm. The size of each cavity was designed to be 400 μm × 1500 μm, with a depth of 10 μm.

The chip of the sensor consists of two H-shaped resonators. The magnitude of the applied pressure is determined by detecting the frequency signals emitted from these two resonators. The resonators can operate at their optimal performance only when they are successfully vacuum encapsulated.

Consequently, detection of the resonant frequency of the resonators and achieving a relatively high quality factor (Q value) through the circuit are crucial indicators. These indicators enable us to confirm that the fabricated Cavity-SOI has effectively accomplished vacuum encapsulation. The successful implementation of this vacuum encapsulation validates the reliability of the wafer level Au–Si eutectic bonding process. Ultimately, this validation offers substantial evidence for the feasibility of the proposed method for fabricating Cavity-SOI.

### 3.2. Sensor Fabrication

**Figure 5:**
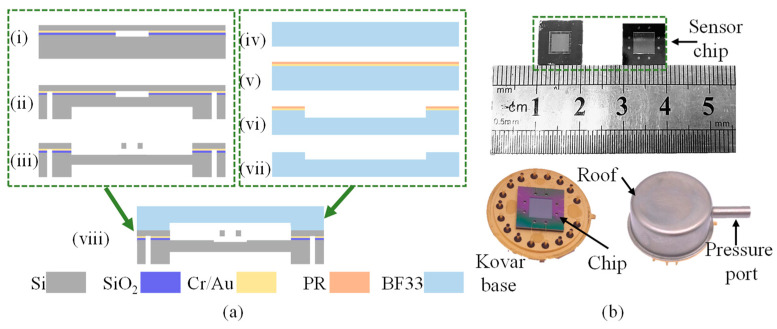
(**a**) Schematic diagram of the fabrication process of the sensor. (**i**) Clean the Cavity-SOI fabricated in Figure 2 using acetone, anhydrous ethanol, and deionized water. (**ii**) Etch the pressure-sensitive diaphragm in the center to a depth of 180 μm and the holes through the substrate layer at the edges on the back of the Cavity-SOI. (**iii**) Etch the resonators and other structures on the device layer. The resonators are positioned directly above the cavities. (**iv**) The BF33 glass is cleaned with concentrated sulfuric acid and deionized. (**v**) Evaporation of a layer of Cr/Au on the cleaned glass surface, followed by spin-coating of a layer of photoresist on the Cr/Au surface. (**vi**) Use the gold etching solution and chromium etching solution to etch the BF3 glass to a depth of 5 μm. (**vii**) Clean the glass, and remove the photoresist and Cr/Au. (**viii**) Bond the Cavity-SOI made in step (**iii**) and the glass cover made in step (**vii**) by anodic bonding. (**b**) Chip dimensions: approximately 10 mm in length and width, with a glass cover on the front and a pressure-sensitive diaphragm and lead holes on the back. And the package of the sensor.

The Cavity-SOI was utilized in the fabrication of RPSs, with the key processes depicted in Figure 5a. Initially, a pressure-sensitive diaphragm and electrode lead holes were formed on the substrate layer of the Cavity-SOI via deep reactive ion etching (DRIE), as shown in Figure 5a(i,ii). Subsequently, resonators, pads, and electrode leads were fabricated on the device layer, as illustrated in Figure 5a(iii). A resonator cover was fabricated from BF33 glass, as shown in Figure 5a(iv–vii), and then it was bonded to the Cavity-SOI through anodic bonding, as depicted in Figure 5a(viii). After the bonding process, the wafer was diced into individual sensor chips. As shown in Figure 5b, the chip has dimensions of 10 mm in both width and length. Subsequently, the chip was packaged using a Kovar base and roof.

**Figure 6:**
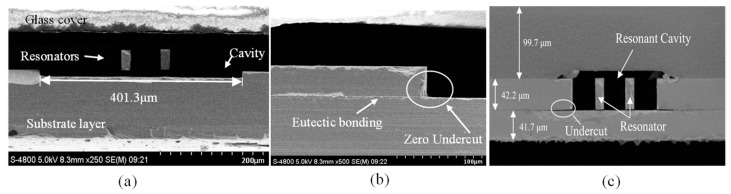
(**a**) SEM photo of the resonator profile, with the cavity located directly below the resonator. (**b**) At the edge of the gold–silicon eutectic bonding interface, compared with (**c**), there is no undercut caused by HF release.

SEM observations showed that the resonator was directly positioned above the cavity, as illustrated in Figure 6a. Moreover, the utilization of Cavity-SOI effectively avoided the formation of undercut during the release of the resonator, as shown in Figure 6b. In contrast, the SEM image of an RPS fabricated with standard SOI and using HF for releasing the resonators exhibited undercut, as depicted in Figure 6c.

### 3.3. Sensor Performance

**Figure 7:**
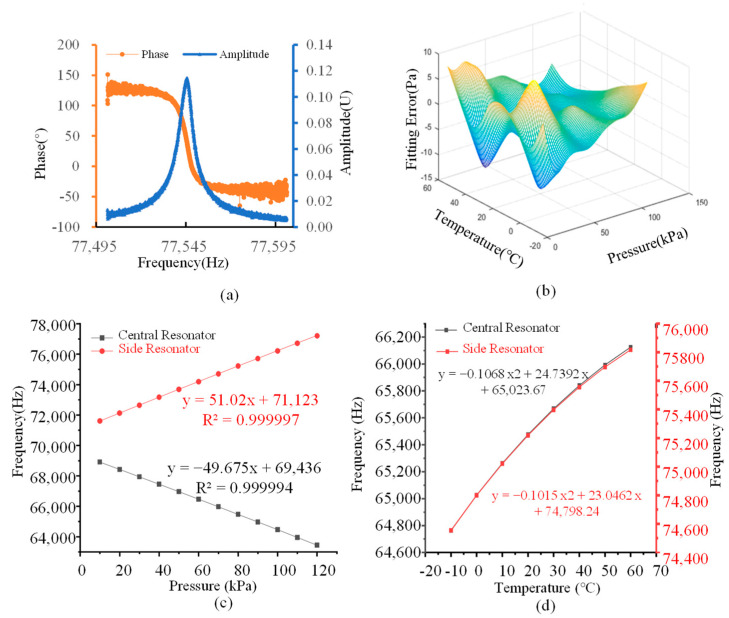
Test results of the sensor. (**a**) Test results of open-loop of microsensor under room temperature and atmosphere pressure, with a resonator Q value of 18,143. (**b**) In the temperature range of −10–60 °C, for the pressure of 1–120 kPa, the sensor is calibrated at different temperatures and pressures, and the error is less than 14 Pa. (**c**) The sensitivity of the side resonator for pressure is 51.02 Hz/kPa with a linearity of 0.999997 and the central resonator’s sensitivity to pressure is −49.675 Hz/kPa with a linearity of 0.999994. (**d**) The temperature sensitivity of the side resonator is 18.155 Hz/°C and the temperature sensitivity of the central resonator is 19.573 Hz/°C.

The sensor output two resonant frequency signals, namely 64,468.02 Hz and 76,224.54 Hz (at 20 °C and 100 kPa). These signals were collected and processed by the circuit to assess the sensor’s performance. We employed a network analyzer to test the amplitude–frequency characteristic curve and the Q value of the sensor, and the results are presented in Figure 7a. A resonator Q value of 18,143 indicates that the resonator is operating in a vacuum environment, which validates the successful implementation of vacuum sealing through wafer-level eutectic bonding and anodic bonding. Among the 52 chips fabricated on the entire wafer, the Q values of the 41 tested chips are greater than 15,000, resulting in a comprehensive yield rate of approximately 78.8%.

The sensor was tested across a temperature range from −10 °C to 60 °C and a pressure range from 1 kPa to 120 kPa. We conducted the calibration of the fabricated sensor by using a fifth-order binary polynomial fitting method, and the pressure p can be expressed as:

(1) $$p=\sum_{i=0}^{5}\sum_{j=0}^{5}k_{ij}f_{1}^{i}f_{2}^{j}$$

where $k_{ij}$ are polynomial coefficients.

As shown in Figure 7b, the polynomial fitting plane had a fitting error of less than 0.012%. The sensitivities of the sensor to temperature and pressure are presented in Figure 7c,d. The pressure sensitivity of the side resonator was 51.02 Hz/kPa, with a linearity of 0.999997. The pressure sensitivity of the central resonator was −49.675 Hz/kPa, and its linearity was 0.999994. The temperature sensitivity of the resonators is depicted in Figure 7d. As we can see, the relationship between temperature and frequency is not strictly linear, which is mainly caused by the differences in the coefficients of thermal expansion of different materials. The temperature coefficient of this sensor is influenced by the glass cover as well as by the different types of materials introduced through the eutectic bonding.

Based on the test results, one resonator exhibited positive pressure sensitivity, whereas the other showed negative pressure sensitivity. Notably, both resonators demonstrated positive temperature sensitivity. As a result, we opted for the differential output mode to enhance the sensor’s sensitivity while minimizing the influence of temperature variations and drift on the sensor’s performance.

## 4. Conclusions

This paper presents a fabrication method for forming Cavity-SOI. The method uses pre-etched cavities, wafer-level low-temperature eutectic bonding, and CMP processes. This enables the customization of both the depth and size of the cavities, thus providing enhanced design flexibility for MEMS devices.

In the fabrication of Cavity-SOI, wafer-level gold–silicon eutectic bonding is utilized. The bonding strength of this process exceeds 1.96 × 10^7^ Pa, and it allows for the successful achievement of vacuum packaging. After applying the CMP process to Cavity-SOI, the surface roughness can be reduced to as low as 1 nm.

Through the fabrication of the RPS, it has been verified that Cavity-SOI can indeed reduce the number of manufacturing process steps. Additionally, it effectively solves the undercut problem that is typically caused by the release process using HF.

Although the method for fabricating Cavity-SOI is a customized approach that requires design considerations based on the specific devices being fabricated, it still offers significant feasibility for the design of more complex MEMS devices. Moreover, it can be integrated into well-established device manufacturing processes, which helps to simplify the overall fabrication workflow.

## Data Availability

The original contributions presented in the study are included in the article, further inquiries can be directed to the corresponding author.
